# Surface and Bulk Oxygen Kinetics of BaCo_0.4_Fe_0.4_Zr_0.2−X_Y_X_O_3−δ_ Triple Conducting Electrode Materials

**DOI:** 10.3390/membranes11100766

**Published:** 2021-10-05

**Authors:** Jack H. Duffy, Yuqing Meng, Harry W. Abernathy, Kyle S. Brinkman

**Affiliations:** 1 Department of Materials Science and Engineering, Clemson University, Clemson, SC 29634, USA; jhduffy@g.clemson.edu (J.H.D.); yuqingm@clemson.edu (Y.M.); 2 National Energy Technology Laboratory, Morgantown, WV 26505, USA; Harry.Abernathy@netl.doe.gov

**Keywords:** triple ionic-electronic conductor, oxygen permeation membrane, electrical conductivity relaxation

## Abstract

Triple ionic-electronic conductors have received much attention as electrode materials. In this work, the bulk characteristics of oxygen diffusion and surface exchange were determined for the triple-conducting BaCo_0.4_Fe_0.4_Zr_0.2−X_Y_X_O_3−δ_ suite of samples. Y substitution increased the overall size of the lattice due to dopant ionic radius and the concomitant formation of oxygen vacancies. Oxygen permeation measurements exhibited a three-fold decrease in oxygen permeation flux with increasing Y substitution. The DC total conductivity exhibited a similar decrease with increasing Y substitution. These relatively small changes are coupled with an order of magnitude increase in surface exchange rates from Zr-doped to Y-doped samples as observed by conductivity relaxation experiments. The results indicate that Y-doping inhibits bulk O^2−^ conduction while improving the oxygen reduction surface reaction, suggesting better electrode performance for proton-conducting systems with greater Y substitution.

## 1. Introduction

Mixed ionic-electronic conductors (MIECs) are utilized in a wide range of applications in intermediate to high temperature systems, from protonic ceramic fuel cells (PCFCs) to oxygen separation and catalysis [1,2,3]. Recently, a subset of MIECs known as triple ionic-electronic conductors (TIECs), predominantly electron hole conductors, but also proton and oxygen ion conductors, have emerged as a promising class of materials, especially as cathodes for PCFCs due to their excellent oxygen reduction reaction (ORR) activity and ease of synthesis [4,5,6]. The conduction of these three species is particularly useful to extend the range of active sites from the electrolyte-electrode interface to the entire surface of the electrode [7].

Despite their strong performance metrics in PCFC applications, bulk properties of TIECs are relatively unknown, especially surface interactions and bulk conductivity of protons and oxygen ions. Historically, measurements for cathode materials focused on characterization in its microstructural, porous architecture, often using electrical impedance spectroscopy (EIS) and power density measurements. These methods provide useful information about ORR kinetics across samples [8], while changes in processes in samples of the same material can be successfully identified [9]. However, it may be difficult to deconvolute these data in different materials as the result of either microstructural effects or material effects. It is therefore pertinent to understand how a material behaves in its dense, nonporous form to understand further how it behaves in functional devices.

Recent work has pushed toward better characterization of bulk TIEC properties, further probing the mechanisms which enable strong surface kinetics and high ionic conductivity in these materials. Electrical conductivity relaxation (ECR) is a prominent method which allows for relatively simple measurements to probe the oxygen reduction kinetics of cathode materials [10,11,12,13,14]. ECR has also recently been used to probe the kinetics of other surface behaviors, such as proton uptake and interactions with water [15,16,17]. Measuring ionic conductivity in TIECs, however, is more challenging, as most known materials in this class are predominantly electron conductors, due to their high doping concentration of multivalent elements, such as Co, Fe, Ni, and Mn [18,19,20]. High electronic conductivity renders traditional methods such as EIS, often used for predominantly ionic conductors, ineffective. Instead, permeation is a widely used technique to probe the ionic conductivity in dominant electronic conductors such as MIECs and TIECs [21,22,23]. Zhou et al. recently utilized ECR and permeation methods to report a new cathode material Sr_2_Sc_0.1_Nb_0.1_Co_1.5_Fe_0.3_O_6−δ_ (SSNCF) with both performance data and bulk property data compared to a well-known material Ba_0.5_Sr_0.5_Co_0.8_Fe_0.2_O_3−δ_ (BSCF) [24]. Despite recent developments, there remains a large literature gap in bulk property data for many claimed triple-conductors. 

Among these TIECs, BaCo_0.4_Fe_0.4_Zr_0.1_Y_0.1_O_3−δ_ (BCFZY0.1) is one of the most widely studied state-of-the-art single-phase cathodes. Aside from strong BCFZY0.1 performance data on PCFCs and SOFCs, its fundamental surface kinetics [15,16,25] and bulk conductivity are well-studied. Multiple variants of BCFZY0.1 have been synthesized and characterized to compare with the base system. A-site-deficient BCFZY0.1 materials have been synthesized, showing improved hydrogen evolution reaction (HER), oxygen diffusion, and hydration parameters from its original structure [26,27]. A B-site deficient variant, Ba(Co_0.4_Fe_0.4_Zr_0.1_Y_0.1_)_0.95_O_3-δ_ (BCFZY0.95), was also synthesized, exhibiting improved proton conduction and ORR kinetics [28,29]. Liang et al. created a Ni-substituted variant, Ba(Co_0.4_Fe_0.4_Zr_0.1_Y_0.1_)_0.95_Ni_0.05_O_3-δ_ (BCFZYN), with improved ORR kinetics and ionic conductivity [30]. These previous results show that BCFZY0.1 can be tuned through doping and deficiency strategies with some measured success.

BCFZY0.1 was initially derived from BaCo_0.4_Fe_0.4_Zr_0.2_O_3−δ_ (BCFZ) [31], as yttrium was substituted into the materials system to impart more proton conductivity and improve ORR kinetics [1,32,33]. The specific composition was chosen based on EIS measurement of the area specific resistance of symmetric cells comprised of different Zr to Y ratios [1]. While the resulting composition was a better performing cathode than BCFZ, the underlying properties of Y substitution in BCFZ remained unstudied.

To better understand how to design TIEC materials for electrodes, including single-phase and multiphase systems, it is paramount to understand the tunability of state-of-the-art, model materials, such as BCFZY0.1. In this work, the oxygen surface kinetics and bulk conductivity are studied for the BaCo_0.4_Fe_0.4_Zr_0.2−X_Y_X_O_3−δ_ (BCFZY_X_, X = 0, 0.05, 0.1, 0.15, 0.2) materials system. It is shown that a tradeoff occurs as Y is substituted into the system, with a small decrease in bulk oxygen ion conductivity and an order-of-magnitude increase in oxygen surface exchange kinetics with increasing Y concentration. This work begins to probe the effect of yttrium in the BCFZY_X_ system and lays the foundation for future work to understand the important factors which make high performance electrode materials.

## 2. Materials and Methods

### 2.1. Preparation of BCFZY_X_ Precursor Powders

All compositions of BCFZY_X_ precursor powders were prepared by a modified Pechini (citric-acid) method [1]. Stoichiometric amounts of Ba(NO_3_)_2_ (Alfa Aesar, 99+%), Co(NO_3_)_2_∙6H_2_O (Alfa Aesar, >98.0%), Fe(NO_3_)_3_∙9H_2_O (Alfa Aesar, >98%), ZrO(NO_3_)_2_ solution (Sigma-Aldrich, ≥99%, 35 wt.% in dilute nitric acid), and Y(NO_3_)_3_∙6H_2_O (Alfa Aesar, 99.9%) were dissolved in deionized water in appropriate molar ratios. The obtained solution is mixed with citric acid (Sigma-Aldrich, ≥99.5%) and EDTA (Alfa Aesar, 99.4+%) with a citric acid to EDTA to metal-ion ratio of 1.5:1.5:1. The solution was then adjusted to reach a desired pH value of 9 by adding NH_4_OH (Alfa Aesar, 28.0–30.0% NH_3_) and heated at 80 °C to obtain a dark purple gel through water evaporation. The gel was dried at 150 °C for 48 h to obtain the primary powder. 

### 2.2. Fabrication of Dense BCFZY_X_ Pellets for Characterization

For preparation of the 1 mm thick membranes, the primary BCFZY_X_ powders were calcined at 1000 °C for 8 h in air. After manually grinding, the powders were mixed with binder (5% polyvinyl alcohol in water) and uniaxially pressed into pellets of 15 mm diameter under 300 MPa for 2 min. The pellets were buried in calcined powder and subsequently sintered at 1270 °C for 8 h. All sintered samples had relative densities greater than 95%. The sintered pellets were subsequently polished to approximately 1 mm thickness using progressively finer sanding paper grits to achieve a polished, shiny surface to reduce surface effects during characterization. 

The BCFZY0.1 membrane was also tested with a porous surface coating of the same composition. To make this surface coating, the BCFZY0.1 primary powder was pre-calcined at 600 °C for 5 h followed by ball milling in 1-butanol (Fisher Chemical, ≥99.4%) for 7 days. The precursor powder was obtained by drying the ball-milled powder at 500 °C for 5 h. Surface coating paste was prepared by mixing the precursor powder with dispersant (20 wt% solsperse 28000 in terpinol solution) and binder (5 wt% Heraeus V-600 in terpinol solution) in a 15:3:1 weight ratio by manual grinding in a mortar and pestle for 45 min. For the surface coated BCFZY0.1 membrane, the BCFZY0.1 paste was screen printed on both sides of the pellet. The whole structure was fired at 900 °C to form the porous BCFZY0.1|dense BCFZY0.1|porous BCFZY0.1 membrane. 

### 2.3. Characterization

The prepared, sintered membranes were crushed into powders and analyzed by X-ray diffraction (XRD; Ultima IV, Rigaku Americas Corporation, The Woodlands, TX, USA) using Cu/kα radiation (λ = 1.54108 Å), with a scan range from 20–90° and a step size of 0.02°. The microstructures of the membranes and coating layer were analyzed using scanning electron microscopy (SEM; SU6600, Hitachi High-Tech, Tokyo, Japan). 

For oxygen permeation measurements, the BCFZY_X_ membranes were sealed on one side with an alumina tube (O.D. = 0.5 in., I.D. = 0.375 in.) by a ceramic paste (Ceramabond 552, Aremco), followed by additional sealing on the sidewalls of the membrane to reduce sidewall permeation and ensure low leakage. A schematic of the experimental setup is provided in Figure S1. The sealed membrane was heated to 100 °C for 2 h and then 250 °C for 2 h to ensure proper curing of the bond before heating to 550 °C at a ramp rate of 1 °C∙min^−1^. The feed side remained unsealed and was exposed to ambient air inside the furnace. The permeate side was supplied with ultra-high purity helium (He; 99.999%, Airgas) at a rate of 50 mL∙min^−1^. The gas concentrations of O_2_ and N_2_ were measured on the membrane permeate side using a gas chromatograph (GC; Micro GC Fusion, INFICON, Bad Ragaz, Switzerland), with the concentration of N_2_ used to calculate the physical leakage of air from the feed side to the permeate side. The oxygen permeation flux was subsequently corrected using the total measured oxygen on the permeate side minus the calculated physical leakage of oxygen. Measured leakage was less than 3% for all samples. 

Electrical conductivity was measured for all BCFZY_X_ samples using a DC four-point probe method with a digital multimeter (Keithley 2001 Series, Tektronix, Inc., Beaverton, OR, USA) on bar samples fabricated from the sintered pellets. Each pellet was polished to create approximately 12 mm x 5 mm x 1 mm dense bars for measurement. Conductivity was measured both in dry air and in humidified air passed through a room temperature (20 °C) bubbler. In addition, this setup was utilized in an ECR method to determine the surface exchange coefficient, *k_chem_*, and the diffusion coefficient, *D_chem_*. Gas composition was abruptly changed from *p*O_2_ of 0.1 to 0.21 at a flow rate of 225 mL∙min^−1^, and the electrical conductivity changed continuously during the gas switching. The ECR measurement was also performed in both dry and humidified air. 

## 3. Results

### 3.1. Structure and Composition

**Figure 1:**
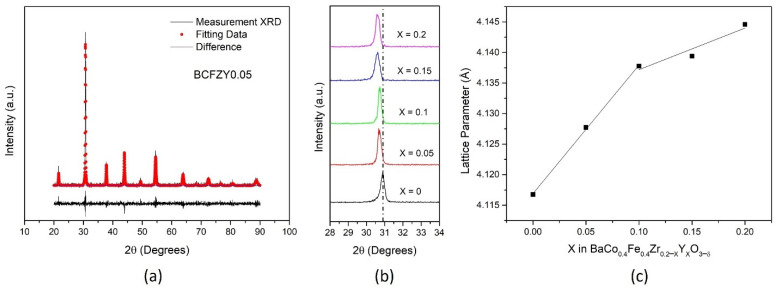
Structural data for (**a**) BCFZY0.05 with Rietveld refinement, (**b**) all compositions for (110) plane, and (**c**) lattice parameter calculated from XRD.

Figure 1a displays the x-ray diffraction pattern for a crushed, sintered pellet of BaCo_0.4_Fe_0.4_Zr_0.15_Y_0.05_O**_3−δ_** (BCFZY0.05) with Rietveld refinement. All compositions, including BCFZY0.05, show a cubic perovskite $\left(Pm\bar{3}m\right)$ phase without any detectable impurities after sintering at 1270 °C for 8 h, and can be seen in Figure S3. The (110) peak is analyzed in Figure 1b, indicating an increased lattice parameter with more incorporated Y. The Y substitution should cause an increase in oxygen vacancies from the original BCFZ material, as written in Kröger–Vink notation as follows:

(1) $$Y_{2}O_{3}+O_{O}^{X}+2Zr_{Zr}^{X}↔2Y_{Zr}^{’}+V_{O}^{**}+2ZrO_{2}$$

Using Rietveld refinement for the measured samples, the calculated lattice parameter for each sample is shown in Figure 1c. Generally, the lattice parameter increased with increased Y substitution, as expected with a larger Y^3+^ cation substitution for the Zr^4+^ cation, consistent with the (110) peak shift from Figure 1b. The lattice parameter was used to calculate parameters which may affect the overall oxygen mobility in the structure, including lattice free volume, critical radius. These data are included in Table S1. 

Sample compositions observed under SEM determined the microstructural differences across compositions. The probed samples show that the average cross-sectional grain size generally increases with Y substitution from 3 um (BCFZ) to 5 um (BCFZY0.05) to 10 um (BCFZY0.1) to 18 um for BaCo_0.4_Fe_0.4_Zr_0.05_Y_0.15_O_3−**δ**_ (BCFZY0.15) and BaCo_0.4_Fe_0.4_Y_0.2_O_3−**δ**_ (BCFY), indicating greater sinterability with greater Y substitution. The grain size is within the same order of magnitude, meaning that there should be no nanostructural effects from grain boundary diffusion of oxygen. In addition, samples were probed using energy-dispersive x-ray spectroscopy (EDX) to confirm the doping of Zr and Y to the system. In all materials, the normalized ratio of Zr:Y was found to be the same as the desired ratio (shown in Table S2). 

### 3.2. Oxygen Permeation Properties

**Figure 2:**
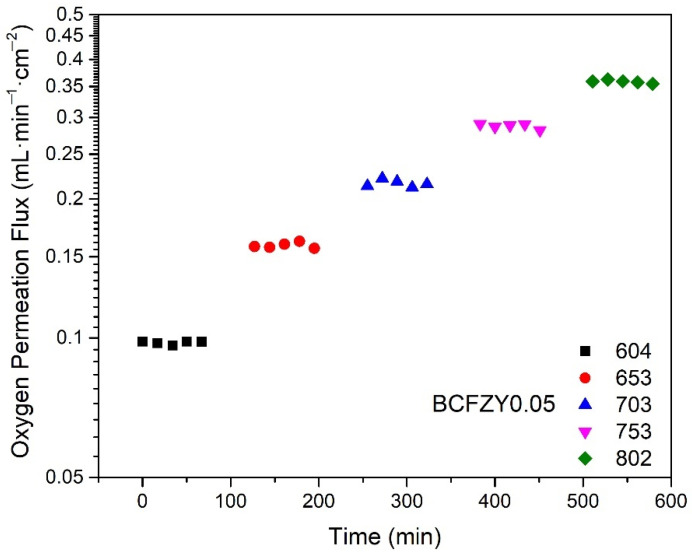
Oxygen permeation flux as a function of measurement time at increasing temperatures for BCFZY0.05, showing stable flux over each one-hour measurement.

A representative figure of the oxygen permeation flux as a function of measurement time is shown in Figure 2 for BCFZY0.05 at temperatures of 600 to 800 °C. After ramping, the temperature is held for 30 min to ensure stabilization of any temperature hysteresis before performing the measurement. Each measurement lasted approximately one hour, with individual gas samples taken in 15-min intervals. The flux remains stable throughout the duration of each temperature. No secondary phase formation or microstructural changes were observed after the permeation measurements as confirmed by SEM/EDX analysis, indicating good material stability in these conditions. In all samples, the final three measurement points at each temperature were averaged as the representative permeation flux for further figures and discussion.

**Figure 3:**
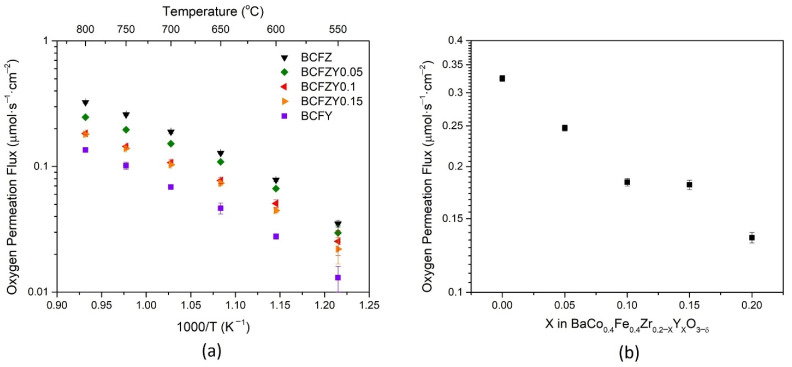
Oxygen permeation flux normalized for 1 mm BCFZY_X_ pellets as a function of (**a**) temperature, and (**b**) Y stoichiometry at 800 °C.

Figure 3a displays the oxygen permeation flux as a function of membrane temperature for all BCFZY_X_ compositions, normalized to 1 mm thickness. Error bars were calculated from measurement propagation error and the standard deviation of the three representative data points. It is observed that as Y substitution increases, the oxygen permeation flux decreases. At 800 °C, as shown in Figure 3b, BCFZ exhibits a flux of 0.485 mL∙min^−1^∙cm^−2^, markedly similar to previously reported literature [34], and continues to decrease with Y substitution until it reaches a plateau for the BCFZY0.1 and BCFZY0.15 compositions, followed by a continued decrease in flux to 0.198 mL∙min^−1^∙cm^−2^ for BCFY. The general trend of decreasing flux with increasing Y is somewhat unexpected, as it was hypothesized that increasing oxygen vacancies in the material would result in more pathways for oxygen diffusion. Based on the structural data from XRD, the estimated free volume and average metal-oxygen bond energy would also suggest that increasing Y concentration would cause an increase in oxygen mobility. Rather, it is hypothesized that Y substitution distorts the lattice from BCFZ due to its much greater ionic size than Zr, Fe, and Co, thus decreasing perovskite symmetry. The decreasing crystal symmetry may cause larger vacancy-lattice interactions and may help explain the trend for oxygen mobility in this materials system [35]. 

**Figure 4:**
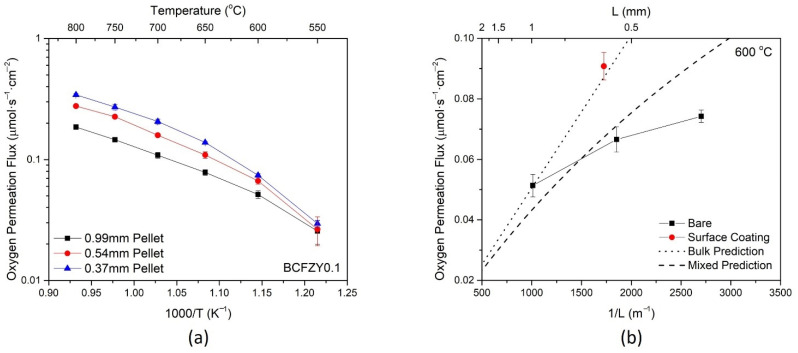
Oxygen permeation flux for the BCFZY0.1 sample (**a**) as a function of temperature, under varying thicknesses, and (**b**) as a function of thickness at 600 °C.

For the BCFZY0.1 composition, three pellets were measured for oxygen permeation flux at thicknesses of 1 mm, 0.54 mm, and 0.37 mm. As shown in Figure 4a, the oxygen permeation increased with decreasing thickness across all temperatures, consistent with the modified Wagner equation below [36]:

(2) $$J_{O_{2}}=-\frac{1}{1+2\left(\frac{L_{C}}{L}\right)}\frac{RT}{4^{2}F^{2}L}\int_{\ln P_{O_{2}}^{’}}^{\ln P_{O_{2}}^{’’}}\frac{\sigma_{el}\sigma_{ion}}{\sigma_{el}+\sigma_{ion}}d\ln P_{O_{2}}$$

where $J_{O_{2}}$ is the oxygen permeation flux, *L_C_* is the material characteristic thickness, *L* is the membrane thickness, *T* is the temperature, *R* and *F* are the gas and Faraday constants, respectively, and $\sigma_{el}$ and $\sigma_{ion}$ are the electronic and ionic conductivities, respectively. Each thickness also shows a characteristic leveling-off of oxygen flux at higher temperatures. This is due to the increase in L_C_ with temperature, resulting in a material with more surface limited performance [36]. 

A similar phenomenon is seen in Figure 4b, which displays the flux as a function of membrane thickness at 600 °C. The theoretical bulk-controlled and mixed-controlled oxygen permeation is plotted using Equation (2) with experimentally determined values, along with the experimental oxygen permeation for bare and coated samples. It is noted that the experimental J vs. L^−1^ plot is non-linear, with thinner bare membranes leveling and exhibiting fluxes further from the theoretical value. This divergence suggests that the membranes become more limited by surface processes as the thickness decreases. 

By adding the surface coating of the same composition at approximately the same thickness, the permeation flux significantly increases. The full surface coating dataset is available in Figure S4. The addition of the surface coating increases the surface exchange kinetics by increasing the availability of surface sites for oxygen reduction, thereby increasing the oxygen permeation. The surface coated membrane also aligns closely with the theoretical bulk-controlled prediction, affirming that this membrane has less surface limitation than the bare sample at similar thickness. The respective increases in flux due to surface coating and decreasing thickness suggest that the bare BCFZY0.1 membranes are controlled by a mixture of surface and bulk processes for the measured thickness range. 

### 3.3. Conductivity

**Figure 5:**
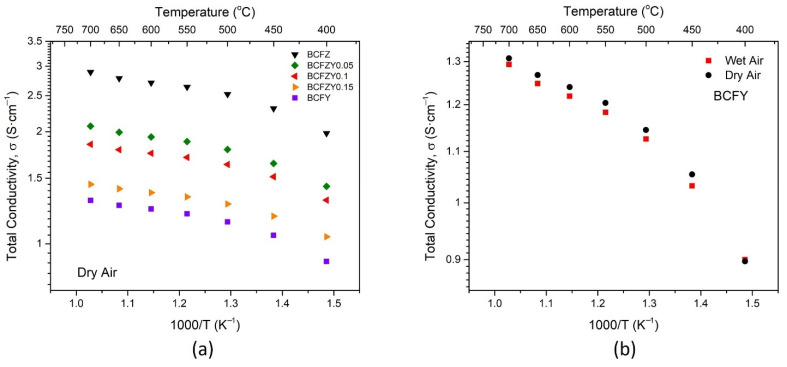
Total conductivity plots as a function of temperature for (**a**) BCFZY_X_ series and (**b**) BCFY under wet and dry atmospheres.

The total conductivity vs temperature plot for all BCFZY_X_ compositions is shown in Figure 5a for dry atmosphere. At 600 °C, the conductivity ranges from 2.71 S∙cm^−1^ for BCFZ to 1.25 S∙cm^−1^ for BCFY, decreasing with increasing Y substitution. This decrease in total conductivity can be somewhat attributed to the increase in lattice constant with Y substitution, as the Y^3+^ ion is larger than the Zr^4+^ ion. The BCFZY_X_ materials also show significantly lower electronic conductivity compared to other triple-conducting cathode materials such as SSNCF [24], BSCF [37,38], and PrBa_0.5_Sr_0.5_Co_1.5_Fe_0.5_O_5+δ_ (PBSCF) [5,39]. 

In wet (2.3% H_2_O) atmosphere, the total conductivity uniformly decreases by about 0.02 S∙cm^−1^ across all samples and temperatures, as seen in Figure 5b for the BCFY composition. This difference in conductivity is attributable to Equation (3), where in redox-active materials, an introduction of humidity leads to a reduction in the hole concentration and conductivity of all compositions at constant oxygen partial pressures [40]:

(3) $$H_{2}O+2O_{O}^{X}+2h^{*}↔2OH_{O}^{*}+\frac{1}{2}O_{2}$$

where $O_{O}^{X}$ and $OH_{O}^{*}$ represents oxygen and hydroxide ions, respectively, on lattice sites, and $h^{*}$ denotes electron holes. In addition, in both wet and dry atmospheres, two characteristic zones of conductivity appear. Below 475 °C, the activation energy E_a_ = 0.11 eV, while above 475 °C, E_a_ = 0.04 eV. This temperature-driven distinction is consistent with previous measurements of BCFZY0.1 [1]. 

From the oxygen permeation measurements, the oxygen ion conductivity can be estimated for all species, as shown in Figure S5. It has been previously shown that BCFZY0.1 exhibits the p-type carrier regime where σ_electronic_ >> σ_ionic_ [25]. Given the similarities in structure, total conductivity, and oxygen permeation, it is reasonable to assume that all BCFZY_X_ compositions follow these p-type regime characteristics. Using this p-type assumption and assuming the membranes are bulk limited ($L_{C}/L$ approaches zero) at 1 mm thickness, Equation (2) can be applied to solve for σ_ion_ using the measured oxygen permeation flux as follows [41]:

(4) $$\sigma_{ion}=\frac{16F^{2}L\cdot J_{O_{2}}}{RT}ln\left(\frac{P_{l}}{P_{h}}\right)$$

where *P_l_* and *P_h_* are the low and high oxygen partial pressures across the membrane. Because the estimates were calculated from the oxygen permeation measurements and the Wagner equation, the same general trend and shape is seen in both the permeation and conductivity data. Relatively small changes in conductivity were observed, as conductivity remained well within an order of magnitude at all temperatures, with approximately a factor of three separating the lowest ionic conductor (BCFY) and the highest ionic conductor (BCFZ) at 800 °C. BCFZY0.1 also exhibited similar conductivity to that in previously reported literature [30]. In air, the ionic transport in relation to electronic transport (t_ion_) remains approximately constant across all samples from 550 to 700 °C, owing to the relative changes in electronic and ionic conductivity across samples. 

### 3.4. Electrical Conductivity Relaxation

**Figure 6:**
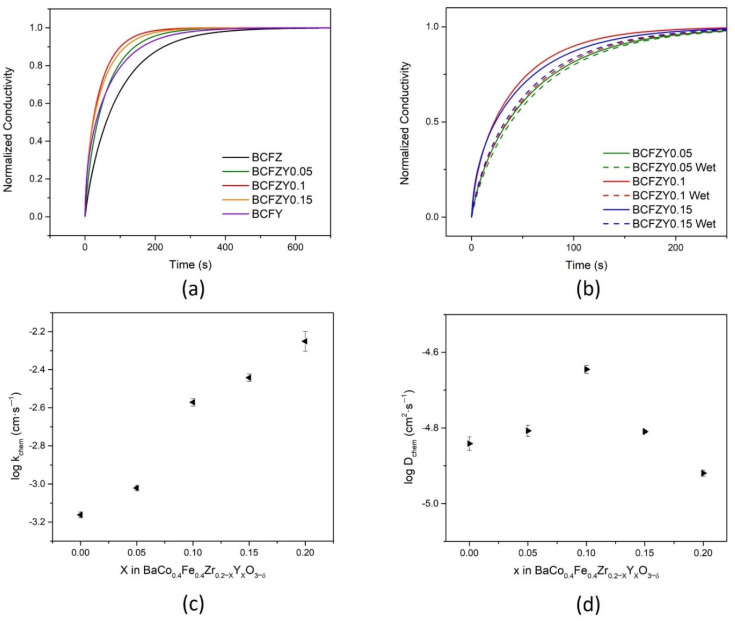
Electrical conductivity relaxation curves at 600 °C. For (**a**) BCFZY_X_ samples and (**b**) BCFZY0.05, BCFZY0.1, and BCFZY0.15 under wet and dry atmospheres; the fitted dry atmosphere curves yield the (**c**) surface exchange coefficient, *k_chem_*, and (**d**) bulk diffusion coefficient, *D_chem_*.

To understand the surface effect of oxygen transport in BCFZY_X_, each composition’s conductivity was measured under rapid gas switching. At high temperatures, oxygen exchange occurs in perovskites according to the following:

(5) $$\frac{1}{2}O_{2}+V_{O}^{**}↔O_{O}^{X}+2h^{*}$$

where $V_{O}^{**}$ represents oxygen vacancies. From Equation (5), the electronic conductivity of each composition is dependent on the oxygen partial pressure. Following a rapid oxidation from 0.1 to 0.21 atm, the conductivity response of each composition is represented in Figure 6a by fitted, normalized conductivity as a function of time at 600 °C. BCFZY0.1 exhibits the fastest response time at around 300 s, increasing in response time as the composition reaches the BCFZ (around 600 s) and BCFY (around 500 s) endmembers. The same gas switch was also performed in humidified atmospheres to simulate PCFC conditions. As shown in Figure 6b for BCFZY0.05, BCFZY0.1, and BCFZY0.15, humidifying air increased the relaxation response time. This increase in response time is likely the result of competition between Equation (5) and the following equation below, as both utilize oxygen vacancies for oxygen and water adsorption, respectively [25]:

(6) $$H_{2}O+V_{O}^{**}+O_{O}^{X}↔2OH^{*}$$

The resultant normalized conductivity curves were fitted using the diffusion equations to determine *k_chem_* and *D_chem_*, specifically using the ECR tool developed by the National Energy Technology Laboratory (NETL) [42]. In Figure 6c,d, the calculated surface exchange coefficient (*k*_chem_) and diffusion coefficient (*D*_chem_), respectively, are displayed as functions of composition. From these data, it is evident that *k_chem_* increases with increasing Y substitution, which agrees with the expectation that an increase in Y substitution increases the oxygen vacancy concentration, resulting in more active sites for surface exchange and reduction via Equation (3). The surface exchange coefficient *k* decreases with the introduction of water to the atmosphere (Figure S6), exhibited by the increase in relaxation time. The diffusion coefficient, *D_chem_*, does not appear to have any clear trend; *k_chem_* has an overall order-of-magnitude increase over the range of Y substitution, while *D_chem_* only changes by about a factor of 2 from its lowest (BCFY) to its highest (BCFZY0.1) values. BCFZY0.1 has the fastest response time of all samples, which may be attributed to the best combination of bulk diffusion with surface exchange. 

These data, coupled with XRD and oxygen permeation data, which showed a decrease in oxygen permeation at 600 °C, suggest that increased oxygen vacancies from Y substitution changes local structuring in the material, which benefits surface reactions, but inhibits bulk oxygen diffusion. The weaker increase in bulk oxygen transport properties is thus impacted by the interplay between a combination of increased oxygen vacancies with the impact on the oxygen vacancy mobility from structural changes. Techniques such as neutron scattering may be used to probe local structure to confirm this hypothesis, presenting a clear path toward understanding these conflicting phenomena. 

**Figure 7:**
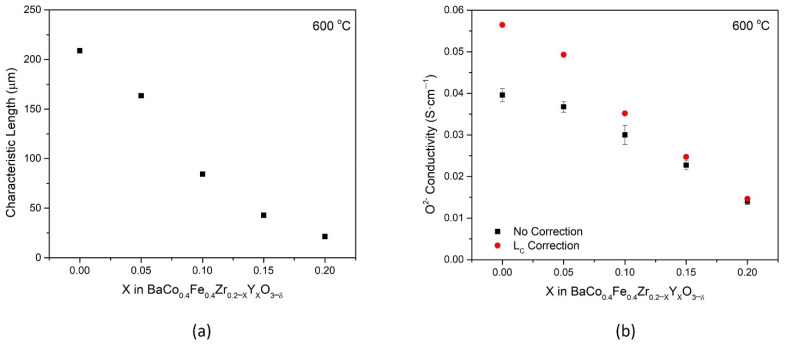
(**a**) The characteristic thickness, L_C_, at 600 °C, calculated from ECR measurements for each BCFZY_X_ composition. (**b**) Oxygen ion conductivity estimated using the Wagner equation with and without a correction for the characteristic thickness.

Using *k_chem_* and *D_chem_* obtained from ECR measurements, the characteristic thickness, L_C_, can be estimated for all compositions at 600 °C. Defined as the ratio of *D* to *k* [21], L_C_ quantitatively expresses the relative control of transport via bulk diffusion or surface exchange. In Figure 7a, the characteristic thickness decreases with increasing Y substitution, from 209 μm for BCFZ to 21 μm for BCFY. This decrease in L_C_ signifies that the materials become more bulk controlled as Y is substituted in the material system as a result of faster surface kinetics. BCFY is identified as a strong candidate for thin-film membranes, due to its low characteristic thickness, and for an infiltration material in fuel cell cathodes because of its superior ORR kinetics.

With experimentally determined L_C_, Equation (2) can be utilized further to estimate the oxygen ion conductivity more accurately at this temperature. Figure 7b displays the oxygen ion conductivity for all BCFZY_X_ samples with and without the correction from characteristic thickness. For compositions with low L_C_, such as BCFY, there is little deviation (about 4%) between the bulk-controlled assumption and the mixed-controlled assumption. However, for compositions with higher L_C_ such as BCFZ, the estimates can deviate by as much as 40%. This deviation highlights the importance of L_C_ to improve the accuracy for permeation as a method of estimating oxygen ion conductivity for triple conducting materials. 

## 4. Conclusions

A suite of BCFZY_X_ samples with different ratios of Zr:Y were probed for oxygen surface kinetics and bulk conductivity. Increasing Y concentrations in this material system increased the lattice parameter of the perovskite structure. In addition, Y substitution resulted in the following across the entire system at 600 °C: (i) an order-of-magnitude increase in oxygen surface exchange coefficient, (ii) a decrease in oxygen permeation by a factor of three, and (iii) a decrease in electronic conductivity by a factor of two. Bulk oxygen ionic conductivity was also estimated for the range of samples, noting the importance of the characteristic thickness on the accuracy of this estimation. It was also determined through the surface coating and varying thickness measurements that the BCFZY0.1 membranes between 1 mm and 0.37 mm were controlled by a mixture of surface and bulk processes. Further research is required on the proton-conducting nature of these materials to better understand underlying reasons for their superior performance as cathode materials.

## Data Availability

All data are contained within the article and its supplementary materials.

## Supplementary Materials

The following are available online at https://www.mdpi.com/article/10.3390/membranes11100766/s1, Figure S1: Oxygen permeation experimental setup, Figure S2: SEM images for BCFZY_X_, Figure S3: BCFZY_X_ X-Ray diffraction, Table S1: Estimated and calculated structural data for BCFZY_X_, Table S2: Stoichiometry of BCFZY_X_ calculated from EDX data, Figure S4: Oxygen permeation flux for bare and surface-coated membranes, Figure S5: Oxygen conductivity as a function of temperature, Figure S6: k_chem_ in wet and dry atmospheres, and Figure S7: Arrhenius plot of oxygen-ion conductivity for BCFZY_X_.

## References

[1] Duan C., Tong J., Shang M., Nikodemski S., Sanders M., Ricote S., Almansoori A., OHayre R. (2015). Readily processed protonic ceramic fuel cells with high performance at low temperatures. Science.

[2] Xu D., Dong F., Chen Y., Zhao B., Liu S., Tade M.O., Shao Z. (2014). Cobalt-free niobium-doped barium ferrite as potential materials of dense ceramic membranes for oxygen separation. J. Memb. Sci..

[3] Tomura Y., Tazawa T., Oikawa I., Takamura H. (2020). Catalytic activity for dissociative oxygen adsorption of Co-based oxides at high temperature evaluated by a modified pulse isotopic exchange technique. J. Mater. Chem. A.

[4] Zohourian R., Merkle R., Raimondi G., Maier J. (2018). Mixed-Conducting Perovskites as Cathode Materials for Protonic Ceramic Fuel Cells: Understanding the Trends in Proton Uptake. Adv. Funct. Mater..

[5] Choi S., Kucharczyk C.J., Liang Y., Zhang X., Takeuchi I., Ji H.-I., Haile S.M. (2018). Exceptional power density and stability at intermediate temperatures in protonic ceramic fuel cells. Nat. Energy.

[6] Papac M., Stevanović V., Zakutayev A., O’Hayre R. (2021). Triple ionic–electronic conducting oxides for next-generation electrochemical devices. Nat. Mater..

[7] Seong A., Kim J., Kim J., Kim S., Sengodan S., Shin J., Kim G. (2018). Influence of Cathode Porosity on High Performance Protonic Ceramic Fuel Cells with PrBa_0.5_Sr_0.5_Co_1.5_Fe_0.5_O_5-δ_ Cathode. J. Electrochem. Soc..

[8] Endler-Schuck C., Joos J., Niedrig C., Weber A., Ivers-Tiffée E. (2015). The chemical oxygen surface exchange and bulk diffusion coefficient determined by impedance spectroscopy of porous La_0.58_Sr_0.4_Co_0.2_Fe_0.8_O_3−δ_ (LSCF) cathodes. Solid State Ionics.

[9] Dierickx S., Weber A., Ivers-Tiffée E. (2020). How the distribution of relaxation times enhances complex equivalent circuit models for fuel cells. Electrochim. Acta.

[10] Wang S., Verma A., Yang Y.L., Jacobson A.J., Abeles B. (2001). The effect of the magnitude of the oxygen partial pressure change in electrical conductivity relaxation measurements: Oxygen transport kinetics in La_0.5_Sr_0.5_CoO_3−δ_. Solid State Ionics.

[11] Li Y., Gerdes K., Diamond H., Liu X. (2011). An improved method to increase the predictive accuracy of the ECR technique. Solid State Ionics.

[12] Li Y., Gerdes K., Liu X. (2013). Oxygen Transport Kinetics in Infiltrated SOFCs Cathode by Electrical Conductivity Relaxation Technique. J. Electrochem. Soc..

[13] Li Y., Gerdes K., Horita T., Liu X. (2013). Surface Exchange and Bulk Diffusivity of LSCF as SOFC Cathode: Electrical Conductivity Relaxation and Isotope Exchange Characterizations. J. Electrochem. Soc..

[14] Kim G., Wang S., Jacobson A.J., Chen C.L. (2006). Measurement of oxygen transport kinetics in epitaxial La_2_NiO_4+δ_ thin films by electrical conductivity relaxation. Solid State Ionics.

[15] Hong T., Lu W., Ren K., Liu T. (2020). The two-fold diffusion process for proton uptake reaction in BCFZY e^−^/H^+^/O^2−^ triple conductor measured by electrical conductivity relaxation. Ionics.

[16] Chen Y., Hong T., Wang P., Brinkman K., Tong J., Cheng J. (2019). Investigate the proton uptake process of proton/oxygen ion/hole triple conductor BaCo_0.4_Fe_0.4_Zr_0.1_Y_0.1_O_3−Δ_ by electrical conductivity relaxation. J. Power Sources.

[17] Ren R., Wang Z., Meng X., Wang X., Xu C., Qiao J., Sun W., Sun K. (2020). Tailoring the Oxygen Vacancy to Achieve Fast Intrinsic Proton Transport in a Perovskite Cathode for Protonic Ceramic Fuel Cells. ACS Appl. Energy Mater..

[18] Ding H., Wu W., Jiang C., Ding Y., Bian W., Hu B., Singh P., Orme C.J., Wang L., Zhang Y. (2020). Self-sustainable protonic ceramic electrochemical cells using a triple conducting electrode for hydrogen and power production. Nat. Commun..

[19] Choi S., Davenport T.C., Haile S.M. (2019). Protonic ceramic electrochemical cells for hydrogen production and electricity generation: Exceptional reversibility, stability, and demonstrated faradaic efficiency. Energy Environ. Sci..

[20] Wang N., Hinokuma S., Ina T., Zhu C., Habazaki H., Aoki Y. (2020). Mixed proton-electron-oxide ion triple conducting manganite as an efficient cobalt-free cathode for protonic ceramic fuel cells. J. Mater. Chem. A.

[21] Chen C.H., Bouwmeester H.J.M., Van Doom R.H.E., Kruidhof H., Burggraaf A.J. (1997). Oxygen permeation of La_0.3_Sr_0.7_CoO_3_−δ. Solid State Ionics.

[22] Balachandran U., Ma B., Maiya P.S., Mieville R.L., Dusek J.T., Picciolo J.J., Guan J., Dorris S.E., Liu M. (1998). Development of mixed-conducting oxides for gas separation. Solid State Ionics.

[23] Balaguer M., Solís C., Serra J.M. (2011). Study of the transport properties of the mixed ionic electronic conductor Ce_1−x_Tb_x_O_2−δ_ + Co (x = 0.1, 0.2) and evaluation as oxygen-transport membrane. Chem. Mater..

[24] Zhou C., Sunarso J., Song Y., Dai J., Zhang J., Gu B., Zhou W., Shao Z. (2019). New reduced-temperature ceramic fuel cells with dual-ion conducting electrolyte and triple-conducting double perovskite cathode. J. Mater. Chem. A.

[25] Meng Y., Duffy J., Na B.T., Gao J., Yang T., Tong J., Lee S., Brinkman K.S. (2021). Oxygen exchange and bulk diffusivity of BaCo_0.4_Fe_0.4_Zr_0.1_Y_0.1_O_3−δ_: Quantitative assessment of active cathode material for protonic ceramic fuel cells. Solid State Ionics.

[26] Ren R., Wang Z., Xu C., Sun W., Qiao J., Rooney D.W., Sun K. (2019). Tuning the defects of the triple conducting oxide BaCo_0.4_Fe_0.4_Zr_0.1_Y_0.1_O_3−δ_ perovskite toward enhanced cathode activity of protonic ceramic fuel cells. J. Mater. Chem. A.

[27] Li X., He L., Zhong X., Zhang J., Luo S., Yi W., Zhang L., Hu M., Tang J., Zhou X. (2018). Evaluation of A-Site Ba^2+^-Deficient Ba_1−x_Co_0.4_Fe_0.4_Zr_0.1_Y_0.1_O_3−δ_ Oxides as Electrocatalysts for Efficient Hydrogen Evolution Reaction. Scanning.

[28] He F., Liang M., Wang W., Ran R., Yang G., Zhou W., Shao Z. (2020). High-Performance Proton-Conducting Fuel Cell with B-Site-Deficient Perovskites for All Cell Components. Energy Fuels.

[29] Kuai X., Yang G., Chen Y., Sun H., Dai J., Song Y., Ran R., Wang W., Zhou W., Shao Z. (2019). Boosting the Activity of BaCo_0.4_Fe_0.4_Zr_0.1_Y_0.1_O_3−δ_ Perovskite for Oxygen Reduction Reactions at Low-to-Intermediate Temperatures through Tuning B-Site Cation Deficiency. Adv. Energy Mater..

[30] Liang M., He F., Zhou C., Chen Y., Ran R., Yang G., Zhou W., Shao Z. (2021). Nickel-doped BaCo_0.4_Fe_0.4_Zr_0.1_Y_0.1_O_3−δ_ as a new high-performance cathode for both oxygen-ion and proton conducting fuel cells. Chem. Eng. J..

[31] Shang M., Tong J., O’Hayre R. (2013). A promising cathode for intermediate temperature protonic ceramic fuel cells: BaCo_0.4_Fe_0.4_Zr_0.2_O_3−δ_. RSC Adv..

[32] Duan C., Hook D., Chen Y., Tong J., O’Hayre R. (2017). Zr and Y co-doped perovskite as a stable, high performance cathode for solid oxide fuel cells operating below 500 °C. Energy Environ. Sci..

[33] Duan C., Huang J., Sullivan N., O’Hayre R. (2020). Proton-conducting oxides for energy conversion and storage. Appl. Phys. Rev..

[34] Tong J., Yang W., Zhu B., Cai R. (2002). Investigation of ideal zirconium-doped perovskite-type ceramic membrane materials for oxygen separation. J. Memb. Sci..

[35] Mogensen M., Lybye D., Bonanos N., Hendriksen P.V., Poulsen F.W. (2004). Factors controlling the oxide ion conductivity of fluorite and perovskite structured oxides. Solid State Ionics.

[36] Hong W.K., Choi G.M. (2010). Oxygen permeation of BSCF membrane with varying thickness and surface coating. J. Memb. Sci..

[37] Chen D., Shao Z. (2011). Surface exchange and bulk diffusion properties of Ba_0.5_Sr_0.5_Co_0.8_Fe_0.2_O_3−δ_ mixed conductor. Int. J. Hydrog. Energy.

[38] Wei B., Lü Z., Huang X., Miao J., Sha X., Xin X., Su W. (2006). Crystal structure, thermal expansion and electrical conductivity of perovskite oxides BaxSr_1−x_Co_0.8_Fe_0.2_O_3−δ_ (0.3 ≤ x ≤ 0.7). J. Eur. Ceram. Soc..

[39] Jiang L., Wei T., Zeng R., Zhang W.-X., Huang Y.-H. (2013). Thermal and electrochemical properties of PrBa_0.5_Sr_0.5_Co_2−x_Fe_x_O_5+δ_ (x = 0.5, 1.0, 1.5) cathode materials for solid-oxide fuel cells. J. Power Sources.

[40] Poetzsch D., Merkle R., Maier J. (2015). Proton uptake in the H + -SOFC cathode material Ba_0.5_Sr_0.5_Fe_0.8_Zn_0.2_O_3−δ_: Transition from hydration to hydrogenation with increasing oxygen partial pressure. Faraday Discuss..

[41] Brinkman K., Iijima T., Takamura H. (2010). The oxygen permeation characteristics of Bi_1−x_Sr_x_FeO_3_ mixed ionic and electronic conducting ceramics. Solid State Ionics.

[42] Na B.T., Yang T., Liu J., Lee S., Abernathy H., Kalapos T., Hackett G. (2021). Enhanced accuracy of electrochemical kinetic parameters determined by electrical conductivity relaxation. Solid State Ionics.

