# Supplementary material for: Surface and Bulk Oxygen Kinetics of BaCo0.4Fe0.4Zr0.2−XYXO3−δ Triple Conducting Electrode Materials
Source: Membranes (Basel). 2021 Oct 5;11(10):766. doi: 10.3390/membranes11100766 (PMC8537876; doi:10.3390/membranes11100766)
Supplement: Supplementary file 1 [file membranes-11-00766-s001.zip › membranes-1401117-supplementary.pdf]

membranes

Supplementary Materials: Surface and Bulk Oxygen Kinetics of $BaCo_{0.4}Fe_{0.4}Zr_{0.2-X}Y_XO_{3-\delta}$ Triple Conducting Electrode Materials

Jack H. Duffy [1,2], Yuqing Meng [1], Harry W. Abernathy [2] and Kyle S. Brinkman [1,2,*]

1 Department of Materials Science and Engineering, Clemson University, Clemson, SC 29634, USA; jhduffy@g.clemson.edu (J.H.D.); yuqingm@clemson.edu (Y.M.)
2 National Energy Technology Laboratory, Morgantown, WV 26505, USA; Harry.Abernathy@netl.doe.gov
* Correspondence: ksbrink@clemson.edu

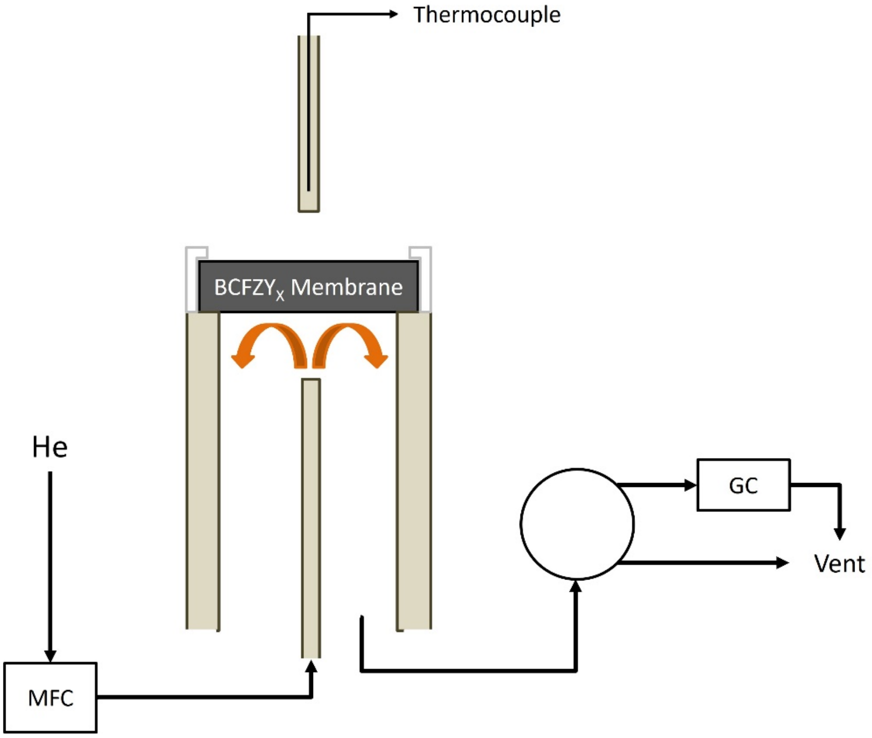

Figure S1. Oxygen permeation experimental setup.

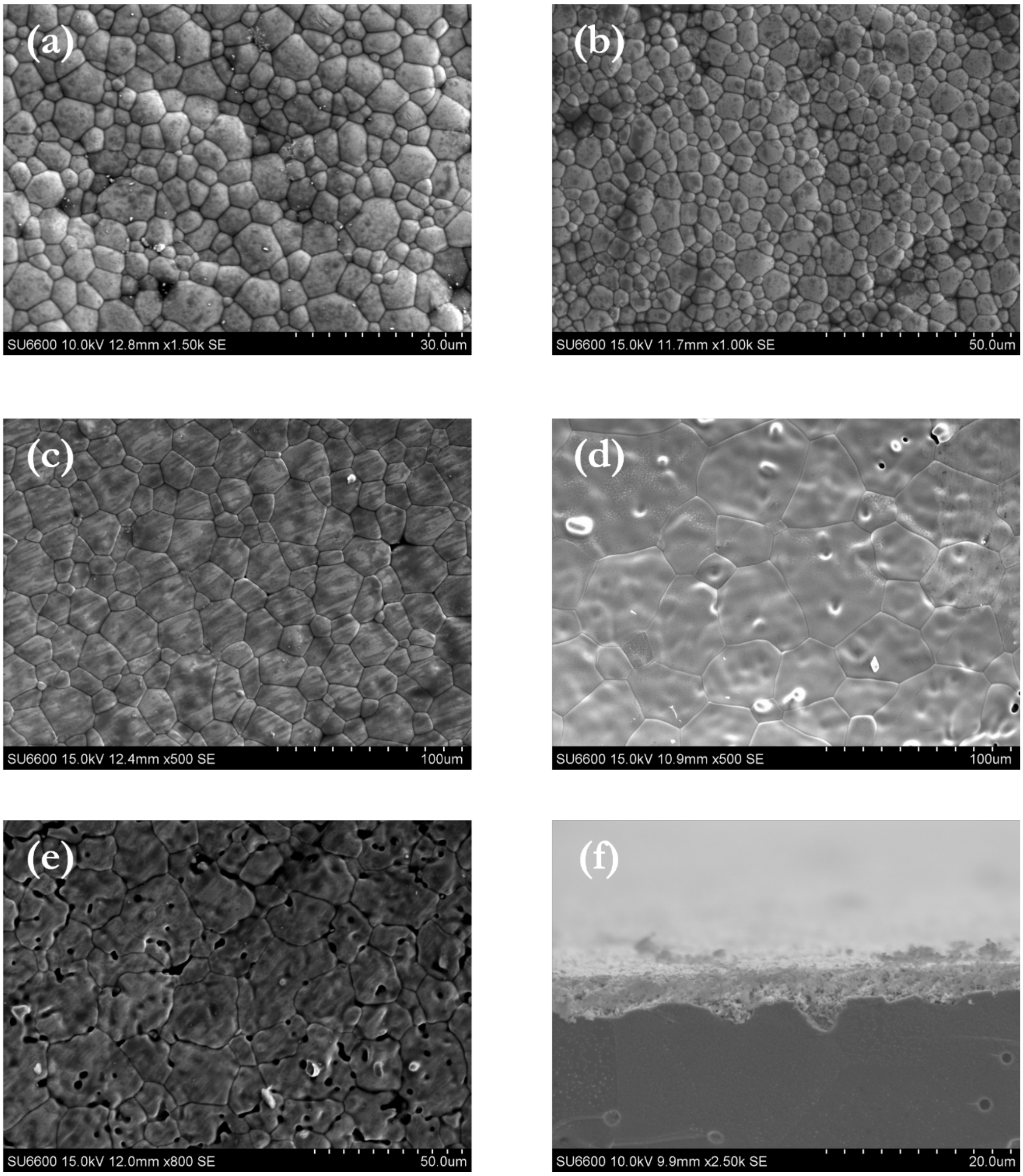

Figure S2. SEM images for BCFZYx, Surfaces for: (a) BCFZ, (b) BCFZY0.05, (c) BCFZY0.1, (d) BCFZY0.15, and (e) BCFY. Cross-section of: (f) BCFZY0.1 with surface coating.

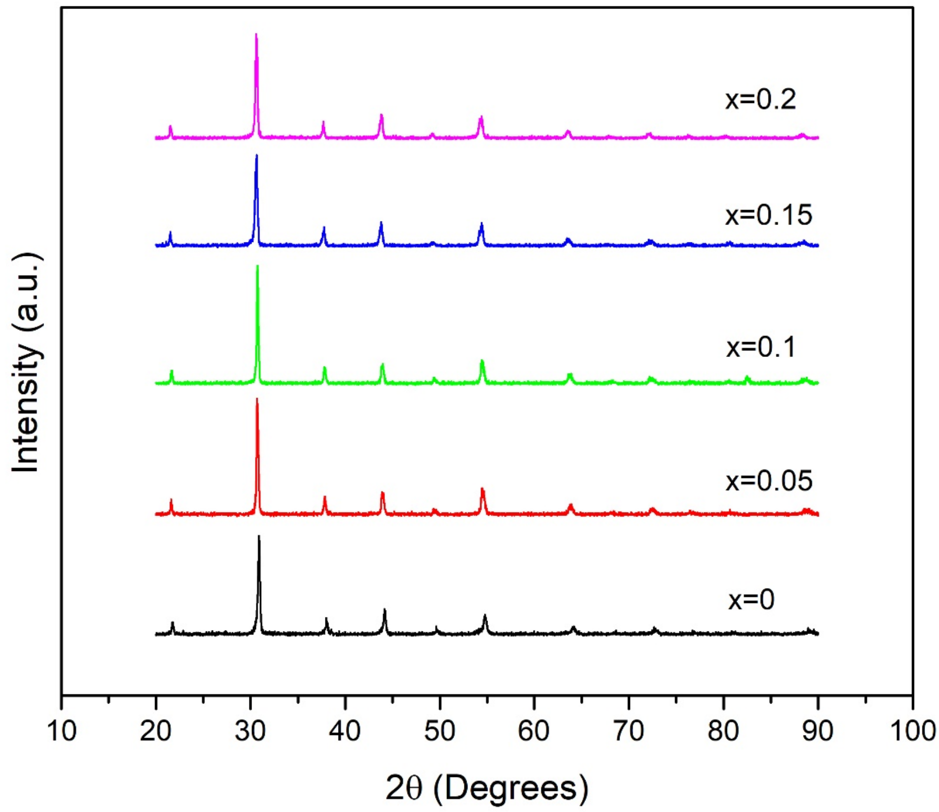

Figure S3. BCFZYx X-Ray diffraction.

Table S1. Estimated and calculated structural data for BCFZYx.

	Estimated Lattice Parameter (pm) [1]	Experimental Lattice Parameter (pm)	Critical Radius (pm) [2]	Free Volume (A^3) [2]	Average Metal-Oxygen Bond Energy (kJ/mol) [3]
**BCFZ**	409.2	411.7	82.48	25.11	-322.09
**BCFZY0.05**	411	412.8	82.74	25.57	-314.12
**BCFZY0.1**	412.8	413.8	82.95	25.98	-306.15
**BCFZY0.15**	414.6	414	82.75	25.98	-298.18
**BCFY**	416.4	414.5	82.7	26.14	-290.21

[1] Lattice parameter and estimated from literature-based ionic radii for given coordination number [1]

[2] Critical radius and free volume estimated using experimental lattice parameter and literature-based ionic radii [1,2]

[3] Average metal-oxygen bond energy estimated from literature values [3].

Table S2. Stoichiometry of BCFZYx calculated from EDX data.

Target Formula	EDX Stoichiometry[1]
$BaCo_{0.4}Fe_{0.4}Zr_{0.2}O_{3-\delta}$	$Ba_{1.29}Co_{0.43}Fe_{0.47}Zr_{0.2}$
$BaCo_{0.4}Fe_{0.4}Zr_{0.15}Y_{0.05}O_{3-\delta}$	$Ba_{1.33}Co_{0.414}Fe_{0.398}Zr_{0.15}Y_{0.047}$
$aCo_{0.4}Fe_{0.4}Zr_{0.1}Y_{0.1}O_{3-\delta}$	$Ba_{1.37}Co_{0.434}Fe_{0.406}Zr_{0.1}Y_{0.097}$
$BaCo_{0.4}Fe_{0.4}Zr_{0.05}Y_{0.15}O_{3-\delta}$	$Ba_{1.31}Co_{0.418}Fe_{0.401}Zr_{0.05}Y_{0.148}$
$BaCo_{0.4}Fe_{0.4}Y_{0.2}O_{3-\delta}$	$Ba_{1.49}Co_{0.55}Fe_{0.55}Y_{0.2}$

[1] EDX stoichiometry normalized to Zr ion in BCFZ, BCFZY0.05, BCFZY0.1, BCFZY0.15, and to Y ion in BCFY.

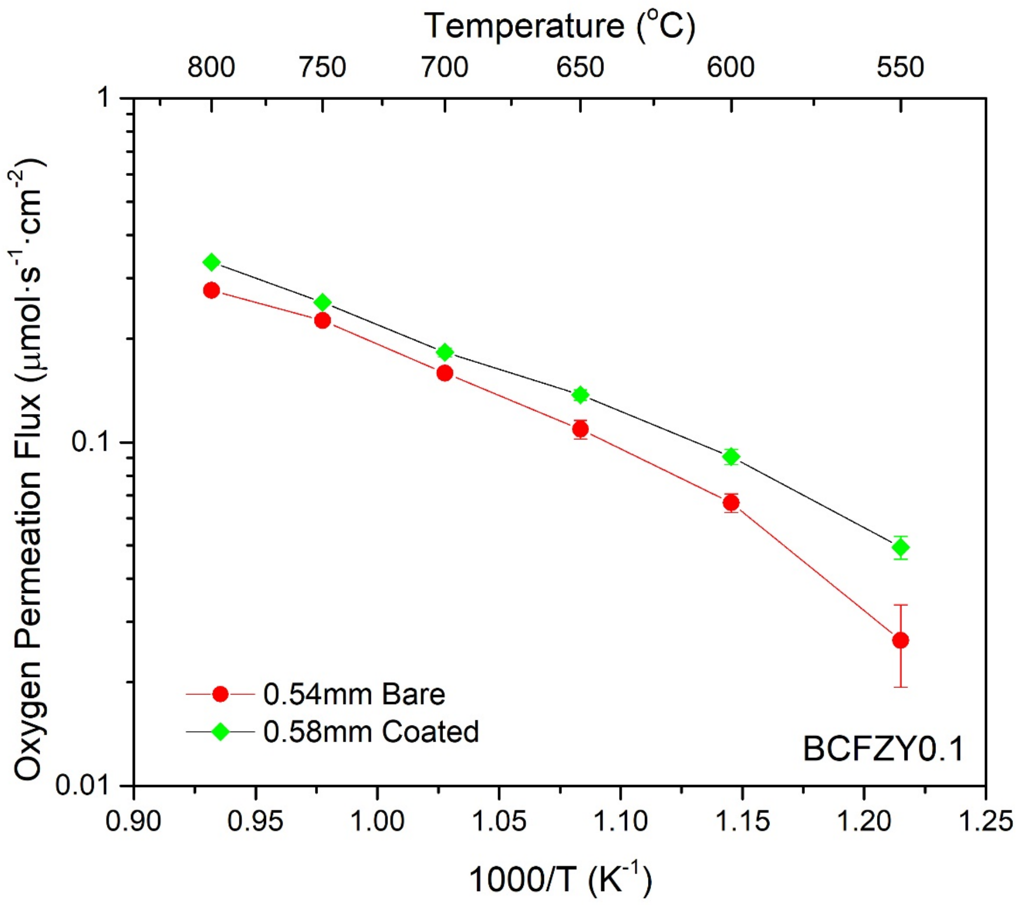

Figure S4. Oxygen permeation flux for bare and surface-coated membranes.

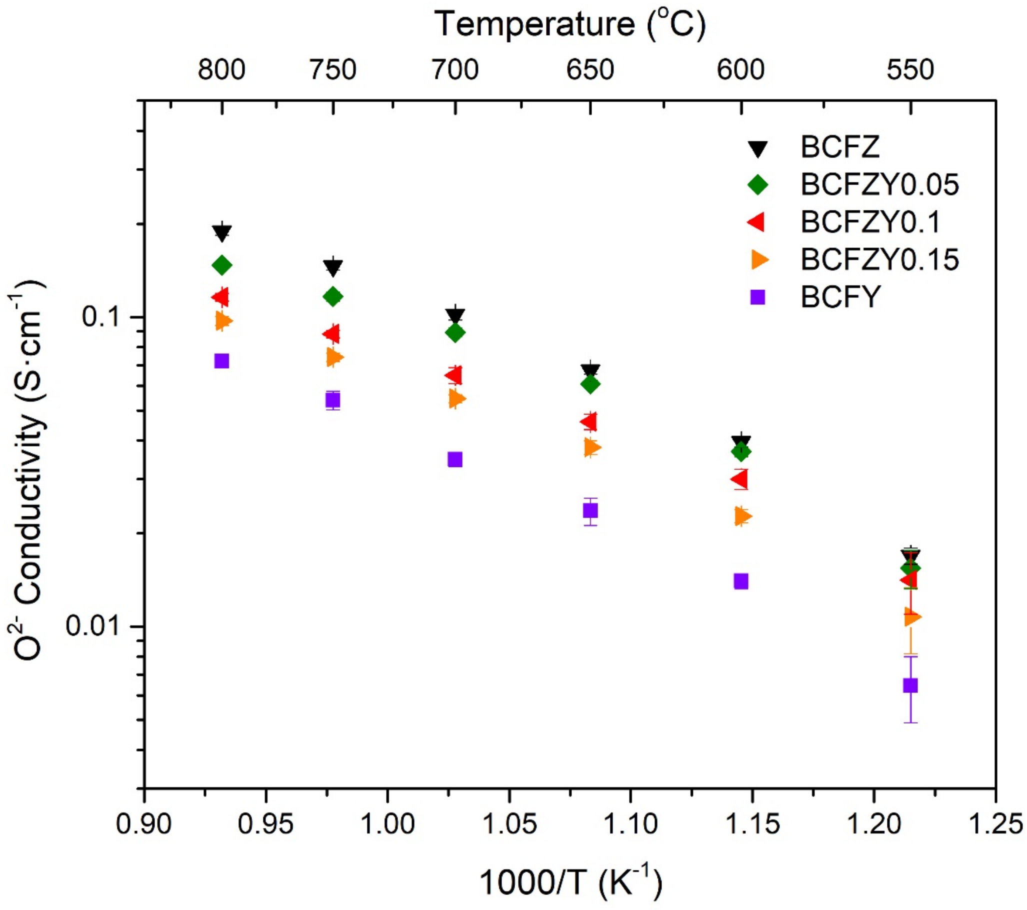

Figure S5. Oxygen ion conductivity as a function of temperature.

Oxygen ion conductivity estimated using the Wagner equation with the assumption that $BCFZY_x$ membranes are bulk-limited.

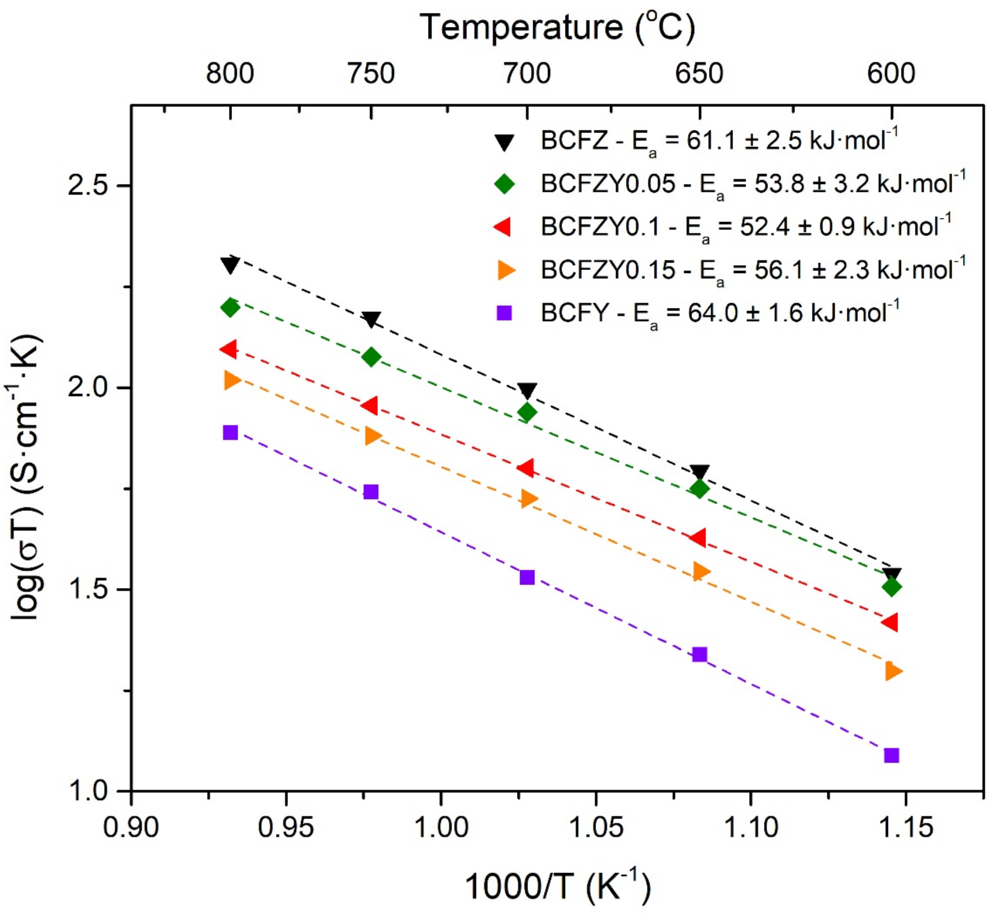

Figure S6. Arrhenius plot of oxygen conductivity for BCFZYx.

References

1. Shannon, R.D. Revised effective ionic radii and systematic studies of interatomic distances in halides and chalcogenides. *Acta Crystallogr. Sect. A* **1976**, *32*, 751–767, doi:10.1107/S0567739476001551.
2. Sammells, A.F.; Cook, R.L.; White, J.H.; Osborne, J.J.; MacDuff, R.C. Rational selection of advanced solid electrolytes for intermediate temperature fuel cells. *Solid State Ionics* **1992**, *52*, 111–123, doi:10.1016/0167-2738(92)90097-9.
3. Klein, S.; Nellis, G. Thermodynamic Properties. In *Thermodynamics*; Cambridge University Press: Cambridge, 2013; pp. 34–91 ISBN 0521195705.
